# Domain-specific Internet use and social participation in older adults: the role of affective attitudes toward digital device use

**DOI:** 10.1007/s10433-026-00930-z

**Published:** 2026-06-23

**Authors:** Nicole Memmer, Sophie Kniepkamp, Anna Schlomann, Laura I. Schmidt, Julia Krönung, Anna-Lena Schubert, Hans-Werner Wahl

**Affiliations:** 1 Network Aging Research, Heidelberg University, Bergheimer Straße 20, 69115 Heidelberg, Germany; 2 University of Hagen, Hagen, Germany; 3 Institute for Educational Sciences, Heidelberg University of Education, Heidelberg, Germany; 4 Institute of Psychology, Heidelberg University, Heidelberg, Germany; 5 Department of Psychology, University of Mainz, Mainz, Germany

**Keywords:** ICT, Technology acceptance, Social activity, Loneliness

## Abstract

Internet use plays an important role in social participation in later life, yet little is known about how different domains of Internet use are linked to social outcomes or which factors shape engagement in these domains. Using an integrative approach, this study examines cognitive and affective attitudes toward Information and Communication Technology (ICT), age, and gender as predictors of social, informational, and entertainment-related Internet use and investigates how these domains relate to subjective (loneliness) and objective (social activity) indicators of social participation. Data were drawn from the baseline assessment of the SMART-AGE randomized controlled trial, comprising 648 community-dwelling adults aged 67–93 years (M = 75.0, SD = 5.63; 52% female). The proposed structural equation model showed good fit to the data. Positive affective but not cognitive attitudes as well as younger age were significantly correlated with all three Internet use domains. Male gender was associated with greater informational and entertainment Internet use, but not with social use. Among the Internet use domains, social use was linked to lower loneliness, informational use was positively associated with social activity, and entertainment use was negatively associated with social activity. Taken together, these findings highlight the importance of affective attitudes in shaping engagement in different forms of Internet use and contribute to a more nuanced understanding of how different forms of Internet use are related to social participation in later life.

As societies around the world undergo demographic changes, social participation of older adults is becoming an increasingly important topic in both academic research and public discourse, given its close links to health, quality of life, psychological well-being, and social capital (Lam et al. 2020; Schutter et al. 2022). Internet use has meanwhile gained considerable relevance for older adults’ maintaining of social participation (Bünning et al. 2023; Hülür and Macdonald 2020). Use of the Internet enables, for example, new forms of communication, access to information, and opportunities for entertainment, all of which may contribute to social participation in later life in various ways. Existing empirical evidence suggests that Internet use can reduce loneliness as well as support various forms of social engagement (Czaja et al. 2018; Yu et al. 2021). However, little is known about how different domains of Internet use, namely social, informational, and entertainment-oriented activities, affect various dimensions of social participation. We posit in this work that differential effects per domain are likely, ranging from enhancing to undermining social participation.

Understanding the associations between different domains of Internet use and social participation is only one side of the coin. Equally important is whether older adults actually engage in online activities that are capable of generating social benefits. To this end, factors such as individual attitudes toward Information and Communication Technology (ICT) and sociodemographic characteristics are crucial for understanding how frequently, and in what ways, older adults use the Internet. Previous research typically has concentrated either on the predictive or outcome side of Internet use and mostly treated Internet use as a monolithic variable.

We offer a combined approach by simultaneously accounting for cognitive and affective attitudes toward ICT as well as age and gender as predictors of different domains of Internet use (social, informational, entertainment) and subjective and objective indicators of social participation as outcome.

## The many faces of Internet use and social participation in later life

Previous research on Internet use and social participation among older adults has generated mixed findings. While some studies observed positive effects, such as increased social engagement, perceived social support, and reduced loneliness (Heo et al. 2015; Szabo et al. 2019; Yu et al. 2021), others have found small, null, or even negative impacts of Internet use on social participation (Meshi et al. 2020; Nowland et al. 2018). We concentrate on two factors that might explain such conflicting findings, both related to the need for a multidimensional view. For one, conflicting findings may be explained by mixing-up of different purposes of Internet use in previous work. Second, social participation itself comprises both subjective components, defined as individuals’ perceptions of social connectedness and feelings of loneliness, and objective components, defined as observable engagement in social activities and social contacts. Distinguishing between these dimensions is crucial for formulating theoretically grounded predictions about how different forms of Internet use relate to social participation in later life.

### A closer look at Internet use in later life

First, *socially framed* Internet use, including digital communication, video calls, or using social network sites, may primarily support subjective aspects of social participation by potentially enhancing emotional closeness despite physical distance in the communication ecology (Hülür & Macdonald 2020). Particularly for older adults, who may face mobility limitations or live geographically separated from loved ones, such digital connections can become a vital source of emotional nearness and support (Nimrod 2020). Szabo et al. (2019) demonstrated that social Internet use among older adults indirectly enhances psychological well-being by reducing feelings of loneliness, a key dimension of subjective social participation. Similarly, Memmer et al. (2026) and Simons (2023) found in their observational studies that frequent digital communication was associated with lower levels of loneliness. Intervention studies further support these findings: A quasi-experimental trial in nursing homes showed that regular video calls with family members significantly reduced loneliness and depressive symptoms while increasing perceived social support among residents (Tsai et al. 2020). In a multisite randomized trial, the use of a tailored computer system for older adults (PRISM) similarly led to reduced loneliness and enhanced social support and well-being after six months of use (Czaja et al. 2018). Taken together, socially oriented Internet use appears to be primarily associated with subjective dimensions of social participation.

Second, *information-centered Internet use,* such as exploring cultural events, checking public transportation timetables, using digital maps, or generally searching for information and services online, may primarily facilitate objective social participation. This is because it helps reduce logistical and informational barriers for engaging in social activities. In addition, information-centered Internet use may serve to improve feelings of preparedness for out-of-home activities and may increase feelings of safety and confidence (Hofer et al. 2019). Studies by Szabo et al. (2019) and Memmer et al. (2025) show that information-centered Internet use is indeed positively associated with objective social participation, including involvement in socially framed leisure activities and forms of community engagement such as volunteering. Further supporting these findings, Schehl (2020) demonstrated that older adults who used the Internet to search for information were significantly more likely to engage in both cultural and community activities. In conclusion, information-centered Internet use is expected to primarily reveal meaningful associations with objective dimensions of social participation, but less so regarding subjective social participation.

Third, *entertainment-related Internet use*, such as streaming movies and series or playing digital games, is becoming increasingly relevant in the everyday lives of older adults (Czaja et al. 2018; Kortmann et al. 2023; Kuoppamäki et al. 2017). However, its effects on social participation seem best characterized as ambivalent (Fan & Yang 2022; Jung & Yu, 2025). On the one hand, digital entertainment can contribute to emotional well-being and a sense of connection, especially when its content is personally meaningful or entertainment is exerted together with others (Lifshitz et al. 2018; Schutter & Brown 2016). On the other hand, researchers have raised concerns about potential negative consequences by referring to the *displacement hypothesis*. As stated by this hypothesis, engaging in digital environments for entertainment purposes may substitute time previously devoted to essential everyday activities, such as participating in social events, community engagement, and face-to-face interactions with close friends and family (Hall & Liu 2022; Nie et al. 2002). Passive digital media consumption like watching videos or movies typically comes with a solitary and non-interactive nature (Putnam 2000; Yang et al. 2021) and has been found to be linked to decreased physical activity, increased loneliness, and lower levels of in-person engagement (Allaire et al. 2013; Fingerman et al. 2022). Applied to later life, the increasing relevance and low threshold of passive digital entertainment may therefore heighten the risk of reduced involvement in offline social activities**,** thereby *negatively* affecting objective social participation.

## Predictors of Internet use among older adults

Taken together, these considerations suggest that different forms of Internet use may relate to distinct dimensions of social participation in later life. However, Internet use itself does not occur randomly. Rather, it is socially structured and shaped by individual characteristics and resources. To fully understand how Internet use may contribute to subjective and objective social participation, it is therefore necessary to examine who engages in which forms of Internet use in the first place. In the following section, we outline key predictors of socially framed, information-centered, and entertainment-related Internet use among older adults.

### Chronological age

With regard to age, previous research consistently shows that older individuals tend to use the Internet less frequently than younger adults. A large-scale analysis by König et al. (2018), based on SHARE data from 17 European countries and adults aged 50 and above, demonstrated a clear negative association between age and regular Internet use. These findings are mirrored in a representative study of older adults in Germany (aged 75 + ; Quittschalle et al. 2020), in which younger age within this cohort was significantly associated with higher Internet use. Yet, it has been rarely examined in previous work how chronological age relates to specific domains of Internet use including social, informational, and entertainment activities.

### Gender

In terms of gender, recent studies suggest that gender differences in Internet use are diminishing over the recent decade. While men continue to report somewhat higher levels of Internet use, differences seem to disappear after controlling for sociodemographic characteristics such as income and education (König & Seifert 2023). Similarly, evidence from the German Ageing Survey shows that between 2014 and 2021 gender gaps in Internet use decreased considerably, with women catching up to men in access and use. Women even overtook men in using the Internet for social contact (Bünning et al. 2023). Taken together, these findings suggest that the gender gap in the domains of social, informational, and entertainment-related Internet use is diminishing to the point of disappearance.

### Attitudes toward ICT

While the historically strong influence of age and gender on Internet use appears to be declining, it remains unclear whether attitudes toward ICT, reflecting individuals’ evaluative stance toward ICT, may also diminish in relevance among older adults (Jeyaraj et al. 2006; Kim et al. 2009; Yang and Yoo 2004; Zhang 2013). Although earlier research has demonstrated that incorporating attitudinal dimensions can significantly improve the explanatory power of models predicting technology use (Mitzner et al. 2010; Yang and Yoo 2004), much of this work is now dated, highlighting the need for updated empirical evidence.

Building on this, classic social psychological research emphasizes that attitudes toward ICT should be differentiated in terms of a cognitive and affective component (Eagly and Chaiken 1993). This distinction acknowledges that evaluations of technology are not solely rational judgments but also involve emotional reactions. Cognitive attitudes refer to individuals’ beliefs and evaluations regarding the overall value of a technology, whereas affective attitudes capture the emotional responses elicited by technology, such as liking or enjoyment (Yang and Yoo 2004).

We argue that it is essential to consider both dimensions simultaneously, as cognitive and affective appraisals may influence technology use in different ways and may play distinct roles in shaping older adults’ engagement with digital tools (Offerman et al. 2023). In fact, according to socioemotional selectivity theory (SST), with increasing age, the focus shifts from informational or performance-related goals toward emotional and social goals (Burnett-Wolle and Godbey 2007; Carstensen 2021; Joshi et al. 2020). Against this established theoretical and empirically proven background, older adults can be expected to increasingly evaluate technologies based on whether they are enjoyable and feel good to use (i.e., affective component), rather than focusing solely on usefulness or efficiency (i.e., cognitive component). Accordingly, it is plausible that affective attitudes are more strongly associated with Internet use among older adults than cognitive attitudes.

## The present study

**Fig. 1:**
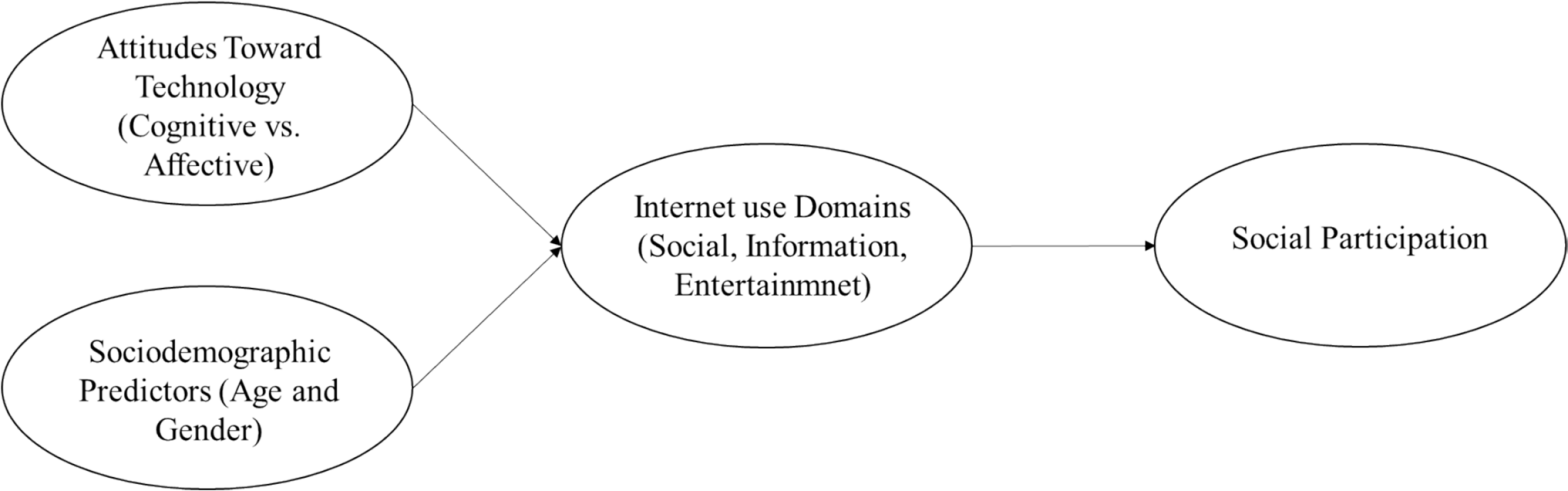
Conceptual model of predictors of Internet use and its domain-specific associations with social participation

The main objective of this study is to test the predictive and outcomes component of Internet use simultaneously in a structural equation model (see Fig. 1).

In terms of the prediction of Internet use, chronological age, gender as well as cognitive and affective attitudes toward ICT are considered. For approaching Internet use in a multidimensional way, three domains are distinguished: social, informational, and entertainment-related Internet use. At the outcome level, we rely on subjective and objective measures of social participation.

Within this network of constructs, we test specific hypothesized relationships using cross-sectional data. First, we focus on predictors of Internet use. We expect that affective attitudes toward ICT will show stronger associations with Internet use than cognitive attitudes, consistent with the notion that emotional engagement may be especially relevant in older age. In light of recent cohort changes in Internet use (e.g., Bünning et al. 2023), we further expect chronological age but not gender as predictive for Internet use. However, when considering all predictors simultaneously, we anticipate that age will be the relatively weakest predictor of Internet use. We also explore whether these patterns hold consistently across the three domains of Internet use: social, informational, and entertainment.

Second, extending the model to outcomes of Internet use, we concentrate on relations among Internet use domains and subjective (loneliness) and objective (social activity) dimensions of social participation. We predict that the three Internet domains will show distinct patterns of association with social participation. Specifically, social Internet use is expected to be negatively associated with loneliness, reflecting enhanced subjective social participation. Information-centered Internet is predicted to be positively associated with greater engagement in socially oriented leisure activities, reflecting increased objective social participation. In contrast, entertainment-oriented Internet use is expected to be negatively associated with objective social participation, possibly due to displacement effects that reduce real-world social engagement. 

## Method

### Study design and sample

The present study is part of the three-armed randomized controlled trial SMART-AGE (Smart Aging in Community Contexts: Testing Intelligent Assistive Systems for Self-Regulation and Co-Regulation under Real-Life Conditions; Schubert et al. 2023; Memmer et al., under review). The study received approval from the ethics committee of the Faculty of Medicine at the University of Heidelberg (S-672/2022).

Individuals aged 67 and older were randomly selected from the residents’ registration offices in two medium-sized cities located in southwestern Germany. Individuals who expressed interest were screened by the study team via phone call. Exclusion criteria included residents of nursing homes, lack of Internet access at home or no prior PC/tablet experience, severe medical conditions, significant visual or hearing impairments, and those working 20 or more hours per week.

For the analyses of this paper, the baseline data of the SMART-AGE study were used, which were collected at the start of the study before any intervention. Data collection took place between April 2023 and August 2024. After exclusion of one participant due to medical reasons, the final sample comprised 648 community-dwelling individuals. The overall sample size was determined based on the preregistered power analyses of the SMART-AGE intervention trial and its three-arm design (Schubert et al., 2023). With respect to statistical power for this study, a minimum sample size of N = 62 would be needed to test the hypothesis of close fit (H0: ε ≤ 0.05, H1: ε ≥ 0.08) for the structural equation model tested in the present study (df = 337), assuming an alpha error of α = 0.05 and a power of 1 – β = 0.80, as suggested by Browne and Cudeck (1992).

On average participants were 75.0 years old (SD = 5.63), and 51.9% were female. The younger age group (67–74 years) included 342 participants, and the older age group (75 + years) included 306 participants. In total, 36.8% of participants lived alone. Overall, the sample showed a relatively high socioeconomic status: 63.4% of participants had a university entrance qualification, and the median household net income fell into category €3,000–€3,499. Regarding health satisfaction, 73.8% of respondents reported being satisfied or very satisfied with their health status. The mean score for depressive symptoms was *M* = 4.21 (*SD* = 2.75) with scores of 5 and above being indicative of mild depressive symptoms. Regarding Internet use, our sample shows lower daily social and informational use (50% vs. 67%; 43% vs. 55%), higher entertainment use (29% vs. 14%), and similar rates of daily gaming (around 13%) compared to the general population of adults aged 60 + in Germany (Rathgeb et al. 2024). In sum, our sample is quite selective in terms of socioeconomic situation, but less so in rates of Internet use.

## Measures

### Predictors of Internet use

In addition to age and gender (0 = female, 1 = male), attitude toward ICT was assessed using items contextualized and adapted from Crites et al. (1994), with answer options following a semantic differential. The construct consists of eight items capturing four cognitive and four affective components of attitudes toward ICT use. The cognitive attitude (4 items; α = 0.91) measures rational evaluations of ICT (e.g., “I think using ICT is a very stupid to very smart idea”), while the affective attitude (4 items; α = 0.91) assesses emotional responses to ICT use (e.g., “Using ICT gives me a very bad to very good feeling”).

### Social participation

Building on Levasseur’s (2022) framework, we aimed to capture key dimensions of social participation. To assess perceived loneliness, we used the German version of the UCLA Loneliness Scale (Döring and Bortz 1993; 20 items; α = 0.93), originally developed by Russell et al. (1980).

Additionally, we measured social activity. Participants reported how often they engaged in 12 different activities over the past 12 months (0 = never to 5 = daily), including meeting friends, volunteering, walking, sports, artistic activities, attending cultural or sports events, participating in political meetings, playing board games, attending classes or lectures, going to the cinema, and going to church (α = 0.65). Although such data must be seen as self-reports, they typically reflect objective behavior quite validly (Levasseur et al. 2022). For all activities except meeting friends and volunteering, participants also indicated whether they performed the activity alone or with others. Each activity was coded with 1 point if it was performed at least monthly and not alone; otherwise, it was coded as 0. A sum score was then calculated, with higher values indicating greater social activity.

### Internet use

We asked participants eleven questions assessing the frequency of their Internet use across various domains. The items were adapted from the German Ageing Survey (DEAS; Stuth et al. 2025) and captured social, informational, and entertainment-related Internet use as conceptually requested for the present study. DEAS items reflect, however, relatively broad and heterogeneous behavioral domains of Internet use, thus reducing internal consistency estimates, when treated as a scale with relatively low item numbers. To account for this, CFA-based composite reliability estimates (omega) were calculated in addition to Cronbach’s alpha (Dunn et al. 2014). The social Internet use scale encompassed 4 items, i.e., contacting friends, acquaintances, and relatives, searching for new social contacts, using social networks, and contributing to blogs or forum discussions (α = 0.54; ω = 0.58). The information seeking scale included 4 items, i.e., searching for general information, checking timetables or travel schedules, seeking health-related information, and using navigation services or maps (α = 0.74; ω = 0.65). The entertainment use scale involved 3 items, i.e., listening to music or watching movies, playing online games, and reading magazines online (α = 0.35; ω = 0.40). Responses ranged from 0 (never) to 5 (daily). Given the importance of the conceptual distinction between social use, information seeking, and entertainment-oriented use of the Internet and the non-availability of established scales in the area, we accepted these in part rather low internal consistency levels.

### Control variables

To account for potential confounding factors, the following control variables were included in the statistical analyses because previous research (e.g., Quittschalle et al. 2020) suggests that they may be associated with both Internet use and social participation: educational level (0 = no degree to 4 = higher education), household net income (1 = less than 500€; 2 = 500–999€ to 11 = more than 4999 €), subjective health status (1 = very dissatisfied to 5 = very satisfied), depressive symptoms (assessed using the PHQ-8 (Kroenke et al., 2009; α = 0.77)), and living situation (0 = living with others, 1 = living alone).

## Statistical analyses

Initially, descriptive analyses were conducted to examine variable distributions, potential differences across age and gender groups, and bivariate correlations. Based on these preliminary insights, we then applied covariance-based structural equation modeling (SEM) with predominantly latent variables. As a sensitivity analysis, the model was also estimated without control variables. The SEM allowed us to simultaneously investigate how cognitive and affective technology attitudes, age, and gender are associated with different domains of Internet use (i.e., social, informational, and entertainment) and, in turn, how these usage domains are related to subjective and objective social participation. The subjective dimension was represented by loneliness, specified as a latent variable based on the UCLA Loneliness Scale using facet-representative parceling. The objective dimension was operationalized using the Social Activity Index.

Analyses were conducted in two steps. First, the measurement model was estimated to confirm the factorial validity of all latent constructs. Based on theoretical considerations and modification indices, several theoretically explainable residual correlations between indicator variables were allowed to account for local dependencies or overlapping content. Second, the full structural model was estimated to examine the hypothesized paths: from predictors to different domains of Internet use (social, informational, and entertainment) and from these domains to social participation.

Full information maximum likelihood estimation was used in both steps to handle missing data. All SEM analyses were performed using the lavaan package in RStudio (Rosseel, 2012). Conventional cutoff values were applied: CFI and TLI ≥ 0.90 indicate acceptable fit, ≥ 0.95 good fit; RMSEA ≤ 0.08 acceptable, ≤ 0.05 good; and SRMR ≤ 0.08 acceptable (Hu & Bentler, 1999).

## Results

### Descriptive statistics

Attitudes toward ICT were assessed using a 0–8 scale. Mean cognitive attitudes were 6.66 (SD = 1.13), and mean affective attitudes were 5.90 (SD = 1.24). Internet use varied across domains, with average frequencies in the mid-range of the 0–5 scale. Informational use was the most frequent (M = 2.55, SD = 0.95), followed by entertainment (M = 2.13, SD = 1.37) and social use (M = 1.78, SD = 1.05). Participants reported lower loneliness (M = 0.82, SD = 0.51; range 0–4) compared to a comparable German sample of older adults assessed with the same UCLA Loneliness Scale (M = 1.4; Memmer et al. 2026). Social activity levels indicated that participants engaged in an average of about three to four different activities monthly with others over the past year (M = 3.49, SD = 1.90; range 0–12).

Descriptive comparisons (Table 1) revealed systematic differences across age and gender groups in predictors of Internet use as well as indicators of social participation. Younger participants (67–74 years) reported more positive technology attitudes (cognitive and affective), more frequent Internet use and higher social participation than those aged 75 +. Gender differences were likewise observable, with women reporting lower loneliness and greater social activity, and men demonstrating higher Internet use alongside more positive affective attitudes toward ICT.

**Table 1:** Descriptive statistics of social participation, Internet use, and attitudes by age and gender

			Age^1^		Gender				
Variable	Range	Total	67–74 (N = 342)	75 + (N = 306)	t test	Female (N = 336)	Male (N = 312)	t test	N
		*M (SD)*	*M (SD)*	*p value*	*M (SD)*	*p value*			
*Predictors of Internet use*									
Attitude (cognitive)	0–8	6.66 (1.13)	6.76 (1.11)	6.54 (1.13)	0.018	6.58 (1.16)	6.75 (1.08)	0.068	599
Attitude (affective)	0–8	5.90 (1.24)	6.03 (1.28)	5.75 (1.18)	0.005	5.81 (1.27)	6.01 (1.20)	0.048	600
*Social Participation*									
Loneliness	0–4	0.82 (0.51)	0.78 (0.52)	0.87 (0.49)	0.037	0.76 (0.50)	0.89 (0.50)	0.002	614
social activity level	0–12	3.49 (1.90)	3.64 (2.01)	3.30 (1.76)	0.027	3.71 (1.91)	3.25 (1.87)	0.003	604
*Internet use*									
Social	0–5	1.78 (1.05)	1.90 (1.02)	1.64 (1.07)	0.003	1.70 (0.91)	1.87 (1.17)	0.045	619
Information	0–5	2.55 (0.95)	2.71 (0.90)	2.35 (0.97)	0.000	2.43 (0.95)	2.67 (0.94)	0.001	616
Entertainment	0–5	2.13 (1.37)	2.24 (1.36)	2.00 (1.37)	0.029	2.03 (1.43)	2.23 (1.30)	0.001	617

*Notes *^*1*^ To facilitate the presentation of descriptive statistics, age was grouped into categories in this table

Table 2 reports bivariate correlations among study variables. First, the age- and gender-related differences reported above were confirmed at the bivariate level. Second, cognitive and affective attitudes toward ICT were strongly and significantly correlated with all three domains of Internet use, indicating that more positive attitudes are associated with higher usage. Third, social and information-related Internet use showed meaningful correlations with all indicators of social participation in the expected direction. In contrast, entertainment-related Internet use was not significantly associated with social participation indicators.

**Table 2:** Intercorrelations of study variables and control variables

		1	2	3	4	5	6	7	8	9	10	11	12	13	14	15	16	17
1	Attitude (cognitive)	1																
2	Attitude (affective)	0.72***	1															
3	Age	-0.13**	-0.11**	1														
4	Male	0.07	0.08*	0.05	1													
5	Loneliness	-0.21***	-0.22***	0.12**	0.13**	1												
6	social activity level	0.06	0.07	-0.1*	-0.12**	-0.37***	1											
7	Life Engagement Test	0.18***	0.19***	-0.1*	-0.01	-0.45***	0.18***	1										
8	Social Support	0.19***	0.21***	-0.2***	-0.04	-0.68***	0.35***	0.32***	1									
9	Social Network	0.12**	0.1*	-0.11**	-0.01	-0.55***	0.38***	0.26***	0.52***	1								
10	Social use	0.31***	0.34***	-0.14***	0.08*	-0.13**	0.12**	0.03	0.16***	0.11**	1							
11	Information use	0.29***	0.27***	-0.26***	0.13**	-0.13**	0.2***	0.11**	0.14***	0.2***	0.35***	1						
12	Entertainment use	0.21***	0.27***	-0.15***	0.07*	-0.02	-0.02	-0.03	0.1*	0.02	0.3***	0.36***	1					
13	Education	0.02	-0.05	-0.06	0.11**	-0.01	0.11**	0.01	0.05	0.06	0	0.23***	0	1				
14	Income	0.15***	0.05	-0.07	0.19***	-0.18***	0.18***	0.09*	0.18***	0.27***	0.04	0.19***	0.1*	0.33***	1			
15	Health	0.08*	0.1*	-0.09*	-0.03	-0.28***	0.11**	0.33***	0.21***	0.18***	0.05	0.03	-0.05	-0.01	0.04	1		
16	Depression	-0.21***	-0.23***	0.14***	-0.09*	0.38***	0.18***	-0.36***	-0.26***	-0.21***	-0.02	-0.04	-0.07	0.05	-0.14***	-0.42***	1	
17	Living alone	-0.03	0	0.18***	-0.3***	0.19***	-0.17***	-0.05	-0.17***	-0.29***	-0.05	-0.12**	-0.07	-0.06	-0.51***	-0.05	0.13**	1

*Notes:* *p ≤ .05, **p ≤ .01, ***p ≤ .001

### Testing a comprehensive model: associations of predictors of Internet use and Internet use and social participation

The model demonstrated an adequate fit to the data: χ^2^ (337) = 826.43, *p* < 0.001, CFI = 0.93, TLI = 0.91, RMSEA = 0.05 (95% CI [0.044, 0.052]), SRMR = 0.06. All item factor loadings were statistically significant, with most exceeding 0.43. The lowest loading within the social use domain was the item assessing contributions to blogs or forums (0.35), while in the entertainment domain the gaming item showed the lowest loading (0.21).

**Fig. 2:**
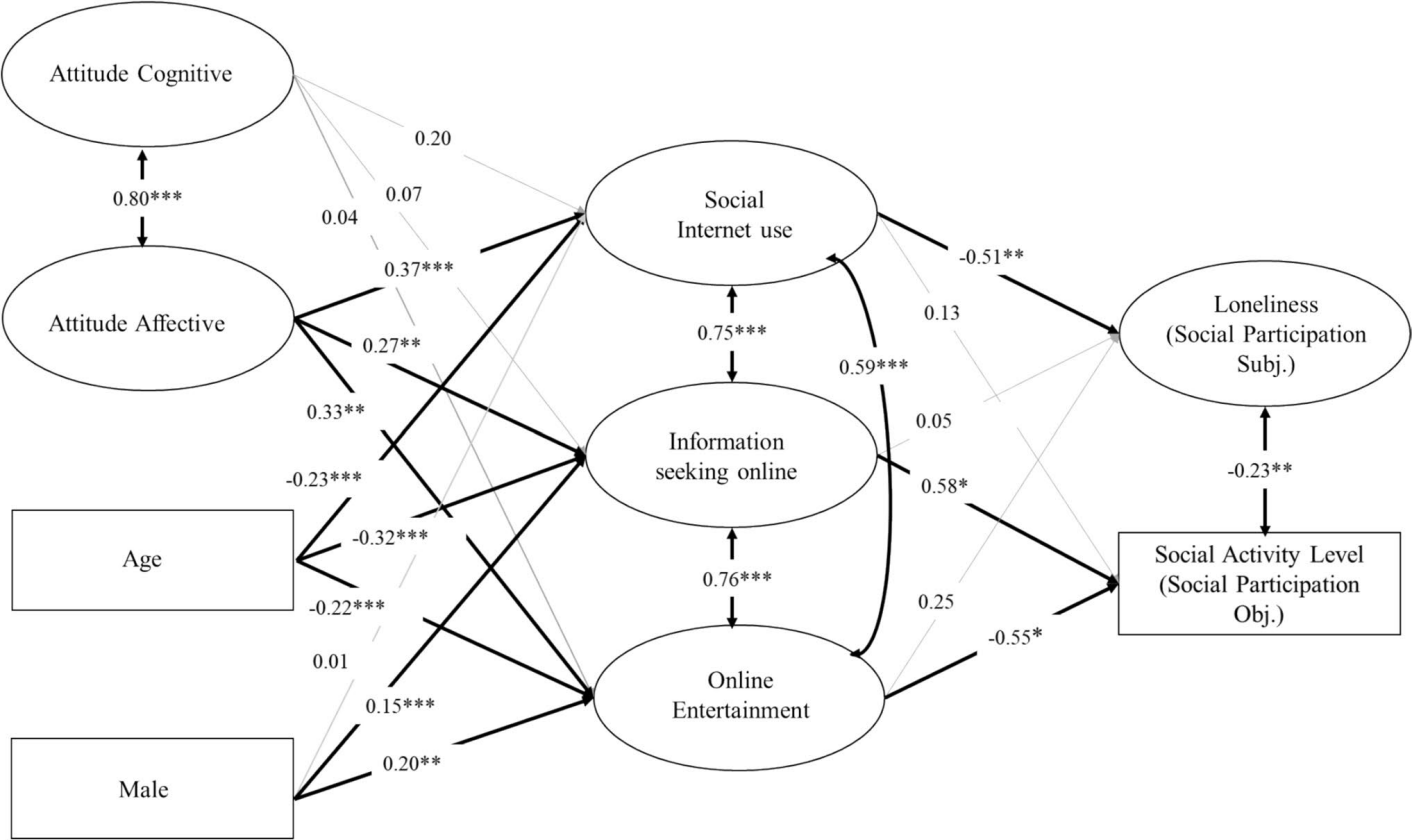
Structural Equation Model of Internet Use and Social Participation (Subj. vs. Obj.) with Control Variables. *Notes:* Residuals for endogenous variables are not shown. *p ≤ .05, **p ≤ .01, ***p ≤ .001. R^2^: Internet use: social = .37; Information = .33; Entertainment = .26; Social Participation: Loneliness = .37; and Social Activity Level = .23

Figure 2 shows the standardized regression coefficients and correlations among the main study variables. Regression paths involving control variables for Internet use and social participation are reported in Table 3. Results were highly comparable when the model was estimated without control variables, suggesting that the main findings were robust to the inclusion of covariates.

**Table 3:** Standardized regression coefficients between control variables, types of Internet use, and social participation

	Education level	Income	Health	Depression	Living alone	Age	Male
*Types of Internet use*							
Social	0.010	-0.058	0.100	0.169	-0.064		
Information	0.213***	0.124*	0.006	0.078	-0.011		
Entertainment	0.057	0.075	-0.159*	-0.055	0.017		
*Social Participation*							
Subjective	-0.029	-0.134*	-0.053	0.404***	0.169**	-0.061	0.176**
Objective	0.006	0.042	-0.073	-0.225***	-0.139*	0.071	-0.182**

First, the affective component of attitudes toward ICT significantly predicted all three Internet use domains, whereas cognitive attitudes did not. Further, higher age was also linked to lower use across all domains, while male gender was only positively related to information and entertainment use. The affective component of attitudes toward ICT showed the relatively strongest association with social and entertainment-related Internet use compared to the other included predictors, whereas higher age showed the relatively strongest association with lower information-related Internet use. The model explained 37.1% of the variance in social Internet use, 33.3% in information use, and 25.8% in entertainment use.

Second, participants who reported more frequent social Internet use also reported lower levels of loneliness. Both information- and entertainment-related Internet use was associated with objective social participation, but information seeking was positively linked to social activity, whereas entertainment-related activities were negatively associated with activity levels. These associations were substantial in magnitude and also were consistent in their associations across the three Internet use domains. However, effect sizes should be interpreted cautiously, as the cross-sectional SEM included multiple correlated self-report constructs that may partly reflect shared method variance. Overall, the Internet use domains explained 36.4% of the variance in loneliness and 23.3% in social activity.

## Discussion

Recent evidence indicates that older adults are using the Internet more frequently, with increases observed even among groups traditionally less active online, such as women (Bünning et al. 2023). Despite these trends, the factors that drive engagement across social, informational, and entertainment-related Internet activities, as well as their relationship with offline social participation, are still not well understood. This study aims to address this gap.

In line with our first hypothesis, our findings indicate that affective, but not cognitive, attitudes toward ICT were associated with engagement in social and entertainment-related Internet activities. These results suggest that positive emotional experiences and enjoyment in digital media use may be more influential than purely cognitive evaluations, which did not significantly predict any of the three usage domains in our model. This is also consistent with previous qualitative research showing that older adults often emphasize enjoyment, pleasure, and emotional satisfaction as major reasons for using technology, frequently outweighing instrumental considerations (Mitzner et al. 2010). A systematic literature review further indicates that older adults’ attitudes are strongly shaped by emotional responses to technology, not merely by rational assessments of usefulness or ease of use (Zhang 2023). These findings also support the assumptions of SST, which posits that older adults prioritize activities that are emotionally gratifying (Carstensen et al., 2021).

Regarding age and gender, older age was consistently negatively associated with all three domains of Internet use, particularly with informational use, whereas men were more likely to engage in informational and entertainment use than women.

Although we build on a selective sample with high levels of education, health, and prior experience with digital devices along all age groups, it is notable that chronological age continues to play a considerable role for Internet use. In other words, cohort effects likely underlying the age effect, which continue to disadvantage the oldest individuals, remain relevant. In some contrast, no significant gender differences were observed in the multivariate model for social Internet use, suggesting that gender has largely lost its predictive role in this domain, but still plays a role in other domains of Internet use (see also Bünning et al. 2023). Taken together, these findings partially support our hypotheses regarding sociodemographic predictors of Internet use: While the diminishing role of gender in social Internet use aligns with our expectations, chronological age remains a stronger and more persistent predictor than anticipated.

Our findings contribute to a better understanding of the mixed evidence regarding the relationship between Internet use and social participation in older adulthood. Importantly, the observed associations differed across Internet use domains, suggesting that links between Internet use and social participation may not be uniform across different types of online engagement.

Social Internet use emerged as the only Internet use domain significantly associated with subjective social participation: Older adults who engaged more frequently in online social activities reported lower levels of loneliness. As hypothesized, social Internet use was negatively associated with loneliness. This finding is consistent with previous research reporting positive associations between digital social interactions and subjective social integration among older adults (e.g., Sims et al. 2017; Szabo et al. 2019). In contrast, informational and entertainment-related Internet use showed no significant associations with subjective social participation**,** which is consistent with our hypothesis that these domains would be less relevant for subjective social participation.

Associations with objective social participation were more nuanced: Informational Internet use was positively associated with the level of social activities, whereas entertainment-oriented use was negatively associated with objective participation. These findings largely support our hypotheses regarding objective social participation: Informational Internet use was positively related to social activity, while entertainment-oriented use showed a negative association. The latter finding is consistent with the displacement hypothesis, which proposes that passive forms of media use, such as streaming, may compete with time spent on offline social activities (Hall and Liu 2022). However, given the cross-sectional nature of the data, the direction of this association remains unclear. It is equally plausible that individuals with lower levels of social activity engage more frequently in entertainment-related online activities.

Overall, these findings underscore that the relationship between Internet use and social participation is multidimensional. Associations with social participation differed across Internet use domains, with more socially or cognitively engaging forms of Internet use showing more favorable associations than more passive and non-interactive forms of use.

## Limitations

Despite these contributions, the study has several limitations. First, the present model combines predictors and correlates of Internet use within a single cross-sectional framework. Although this approach allows for an integrated examination of theoretically relevant relationships, some covariates (e.g., health and depressive symptoms) may also be part of more complex reciprocal processes involving Internet use and social participation. Consequently, the observed associations should not be interpreted causally. It is equally plausible that older adults who are already more socially active engage differently in Internet use, particularly in social and informational online activities, or that both are influenced by common underlying characteristics. Longitudinal studies are needed to clarify the direction of these relationships.

Second, the sample consisted predominantly of well-educated and healthy older adults with basic digital skills, which may limit the generalizability of the findings. In the present sample, affective attitudes toward ICT were consistently associated with Internet use, whereas cognitive attitudes showed no significant associations. However, previous research (e.g., Heart and Kalderon 2013; Hill et al. 2015) suggests that in more vulnerable populations, barriers such as limited digital access, lower digital literacy, or cognitive constraints may play a more prominent role in technology engagement. Consequently, the relative importance of cognitive and affective attitudes toward ICT, as well as the associations between Internet use and social participation, may differ across populations. Future research should therefore examine these relationships in more diverse and less digitally privileged groups of older adults.

Third, some limitations regarding the measurement of Internet use domains should be considered. Although the latent measurement model showed an acceptable to good fit and all factor loadings were statistically significant, the entertainment-related Internet use domain showed comparatively low reliability estimates. This likely reflects that the domain captures a broad range of online activities rather than a highly homogeneous construct. Accordingly, findings related to entertainment-related Internet use should be interpreted with some caution. Future research may benefit from distinguishing between different forms of entertainment-related Internet use to determine whether they have differential associations with social participation and well-being.

Finally, we acknowledge that our measure of objective social participation is based on self-reports rather than actual behavioral or observation-based measures. This may introduce reporting biases and limit the extent to which our findings capture true objective social participation.

## Practical implications

The findings have several practical implications for promoting meaningful Internet engagement among older adults. First, interventions and training programs may benefit from focusing not only on technical skills or access but also on fostering positive emotional experiences and enjoyment in digital activities, as affective attitudes were consistently associated with Internet engagement across domains. Encouraging enjoyable and emotionally satisfying experiences may increase the likelihood that older adults actively use digital tools.

Second, the observed associations differed across Internet use domains, suggesting that different forms of online engagement may be linked to social participation in different ways. In particular, social and informational Internet use showed more favorable associations with social participation than entertainment-oriented use. These findings may help inform the design of future interventions and should be further examined in longitudinal and experimental research.

Finally, interventions should take age-related differences into account. Older adults, especially those from cohorts with less prior exposure to digital technologies, may require additional support to access, navigate, and feel comfortable with digital tools.

## Conclusions

This study highlights that different forms of Internet use are associated with social participation in different ways among older adults. Specifically, individuals who reported more social and informational use also reported greater subjective and objective social participation. In contrast, entertainment-oriented use showed less favorable associations with social participation. Affective attitudes toward ICT emerged as key predictors of Internet use, highlighting the potential importance of enjoyment and positive emotional experiences. Age continues to influence Internet use patterns, while gender plays a more limited role. Overall, our findings suggest that socially and cognitively engaging Internet activities may be more closely linked to social participation than more passive forms of online engagement. Future research should examine these relationships longitudinally to better understand their directionality and underlying mechanisms.

## Data Availability

The data and analysis script used in this study are part of a larger research project and are therefore not publicly available at the time of submission. To ensure transparency and reproducibility during the review process, a subset of the data relevant to this study, along with the corresponding analysis scripts, has been made available to the reviewers via a view-only Open Science Framework (OSF) link (https://osf.io/7jd5k/overview?view_only=6ee88b4f32994758a9f41b634db9d545). The full dataset will be released publicly alongside the other project components at a later stage. We expect this release before possible publication of this manuscript in which case we would update the links accordingly.
